# The Effects of Metabolic Bariatric Surgery on Intra-pancreatic Fat Deposition and Total Pancreas Volume: a Systematic Review and Meta-analysis

**DOI:** 10.1007/s11695-025-07778-9

**Published:** 2025-03-17

**Authors:** Yaochen Wang, Yutong Liu, Maxim S. Petrov

**Affiliations:** School of Medicine, University of Auckland, Auckland, New Zealand

**Keywords:** Metabolic bariatric surgery, Endoscopic bariatric therapy, Fatty pancreas disease, Intra-pancreatic fat deposition, Total pancreas volume

## Abstract

Bariatric procedures are common interventions for weight loss and metabolic improvements; yet, their effects on the pancreas—a key metabolic organ—are largely unknown. This systematic review aimed to summarise data on changes in intra-pancreatic fat deposition (IPFD) and total pancreas volume (TPV) following metabolic bariatric surgery and endoscopic bariatric therapy. The literature search was conducted in MEDLINE and Embase databases. Studies in humans were included if they reported on changes in IPFD and/or TPV from before to after bariatric procedure. Random effects meta-analysis was conducted. Fourteen studies (all involving metabolic bariatric surgery) met the eligibility criteria. Metabolic bariatric surgery resulted in a mean absolute IPFD reduction of 3.9% (*p* = 0.003) and a mean TPV decrease of 10.7 cm^3^ (*p* < 0.001). The mean relative IPFD reduction after metabolic bariatric surgery was 35.9%. Sleeve gastrectomy led to a higher mean relative IPFD reduction than Roux-en-Y gastric bypass (39.9% vs. 20.6%), though this difference was not statistically significant. Analysis of follow-up duration showed a mean relative IPFD reduction of 8.8% in patients with follow-ups of less than 3 months. Metabolic bariatric surgery leads to significant reductions in IPFD and TPV. Follow-up should take place no earlier than 3 months for the full benefits of bariatric procedures on the pancreas to manifest.

**Supplementary Information:**

The online version contains supplementary material available at 10.1007/s11695-025-07778-9.

## Introduction

Obesity and related metabolic disorders are one of the most pressing health challenges in the 21^st^ century. The World Health Organisation classified obesity as an epidemic due to the rise in obesity prevalence around the world, with projections that it will affect more than one billion adults globally by the year 2030 [1]. Metabolic bariatric surgery—the two most common types of which are sleeve gastrectomy (SG) and Roux-en-Y gastric bypass (RYGB)—is among the most effective interventions for morbid obesity, resulting in considerable weight loss and improvement in obesity-related metabolic disorders [2]. With the number of surgical interventions for obesity growing dramatically (140,000 in 2003 vs. 720,000 in 2018) [3], metabolic bariatric surgery is a critical armamentarium in the management of morbid obesity. Further, endoscopic bariatric therapy is offered to patients with obesity who are not suitable for metabolic bariatric surgery [4]. Bariatric procedures are known to often result in improvements in glucose control and insulin sensitivity [5]. Although weight loss is the main goal of bariatric procedures, changes in the composition of organs (e.g., the pancreas—a key organ whose functions affect whole-body metabolism) are also important [6].

High intra-pancreatic fat deposition (IPFD) is the most common disorder of the pancreas [6], which may lead to both incident diseases of the endocrine pancreas (e.g., type 2 diabetes) and incident diseases of the exocrine pancreas (e.g., pancreatic cancer, pancreatitis) [7–9]. Around one-fifth of people in general population have high IPFD, as conservatively estimated in high-quality studies using magnetic resonance imaging (MRI) [10, 11]. There has been recent evidence of changes in IPFD following metabolic bariatric surgery [12, 13]. In addition to IPFD, the effect of metabolic bariatric surgery on total pancreas volume (TPV)—a broader indicator of the organ’s composition and morphology—has been investigated [14]. While these studies consistently found a decrease in IPFD, its extent varied considerably between the individual studies [12–14]. These variations might have been attributed to types of surgery, duration of follow-up, and imaging protocols used. In addition, the relationship between IPFD reduction and changes in metabolic markers is currently unclear. Notably, our earlier meta-analysis on the clinical burden of high IPFD showed no statistically significant relationship between IPFD and body mass index [15].

The primary aim of this study was to conduct a systematic review of changes in IPFD and TPV following bariatric procedures. The secondary aims were to assess whether the type of metabolic bariatric surgery influenced changes in IPFD, and to evaluate the variability in IPFD reduction according to follow-up duration as well as imaging modalities used. The tertiary aim was to investigate the interrelationships of changes in IPFD with metabolic parameters.

## Methods

### Eligibility Criteria and Literature Search

A comprehensive search was conducted in the Embase and MEDLINE databases to identify clinical studies in adults (aged 18 or above) that meet the criteria below:

1. Study design – prospective or retrospective cohort studies;
2. Interventions – metabolic bariatric surgery or endoscopic bariatric therapy;
3. Outcome measures – both pre- and post-procedural measurements of IPFD and/or TPV (determined with the use of MRI, magnetic resonance spectroscopy, or computed tomography (CT).

The following search string was developed in consultation with an experienced subject librarian:

1. Bariatric surgery/
2. (“bariatric surgery” or “sleeve gastrectomy” or “gastric bypass” or “Roux* BPD*” or billio* or SAD* or “single-anastamo*” or endoscop* or “bariatric endoscop*” or gastroplast* or balloon* or “metabolic surgery” or “weight loss surgery”).mp. [mp = title, abstract, heading word, drug trade name, original title, device manufacturer, drug manufacturer, device trade name, keyword heading word, floating subheading word, candidate term word].
3. 1 or 2
4. Tomography, X-ray computed/
5. Magnetic resonance imaging/
6. (CT or MRI or “comp* tomography” or “Magnetic resonance imaging” or “MRS” or “MR spectroscopy” or “Magnetic resonance spectroscopy” or “nuclear resonance imaging” or imaging).mp. [mp = title, abstract, heading word, drug trade name, original title, device manufacturer, drug manufacturer, device trade name, keyword heading word, floating subheading word, candidate term word].
7. 4 or 5 or 6
8. (“pancrea* adipose” or “pancrea* fat” or “pancrea* volume” or “pancrea* size” or “pancrea* steato*” or “Ecotopic fat*” or “ectopic adipose”).mp. [mp = title, abstract, heading word, drug trade name, original title, device manufacturer, drug manufacturer, device trade name, keyword heading word, floating subheading word, candidate term word].
9. 3 and 7 and 8

Non-peer-reviewed manuscripts, conference abstracts, technical reports, preprints, opinion pieces, white papers, and theses or dissertations were excluded.

### Selection Process and Data Extraction

The initial screening of all titles and abstracts identified through the database search was conducted to determine eligibility for full-text review. The pre-specified abovementioned eligibility criteria were applied. For studies published in languages other than English, a machine translation tool (Google Translate) was used to translate the full texts into English. Data extraction focused on the following main categories: (1) general information: authors, year of publication, country of study, and title; (2) participant characteristics: age, sex, body composition, associated medical problems; (3) intervention and outcome: type of bariatric surgery, follow-up duration, imaging protocol, changes in the pancreas, metabolic parameters.

### Methodological Quality Assessment

Quality assessment of eligible studies was conducted using the Newcastle–Ottawa scale—a tool designed for evaluating the quality of non-randomised studies in meta-analyses [16]. Three broad aspects were assessed: selection of study groups, comparability of the data, and ascertainment of outcomes of interest. Each study was then scored out of a maximum score of 9. The higher the number, the higher the methodological quality. A study with a score of 9 would have a low risk of bias, a score of 7 or 8 would indicate a moderate risk of bias, and a score of 6 or less would indicate a high risk of bias [16].

### Statistical Analysis

The primary outcomes—changes in IPFD and TPV—were analysed using the mean difference (MD) to quantify the effect size in each study. The pooled MDs were calculated with 95% confidence intervals (CIs) to determine the overall effect. In order to explore the influence of different factors on changes in the outcomes, subgroup analyses were conducted according to the type of metabolic bariatric surgery (SG vs. RYGB), follow-up duration (less than 3 months, 3 months, 6 months, and 12 months or more), and imaging modality (CT vs. MRI). Data on changes in IPFD after metabolic bariatric surgery in relation to baseline IPFD, weight, and metabolic parameters were pooled using the Pearson correlation coefficient and a random effects model with the restricted maximum likelihood estimator. Statistical heterogeneity across studies was assessed using the *I*^2^ statistic, which measures the percentage of total variation across studies attributable to heterogeneity rather than chance. An *I*^2^ value of 25% or less was deemed to denote low heterogeneity, 25% < *I*^2^ < 75%—moderate heterogeneity, and 75% or more—high heterogeneity. A *p* value of less than 0.05 was deemed statistically significant in all analyses. The above analyses were performed using Review Manager (Revman) version 5.4 (The Cochrane Collaboration, London, UK) and Jamovi version 2.5 (Jamovi, Sydney, Australia).

## Results

### Study Characteristics

**Fig. 1:**
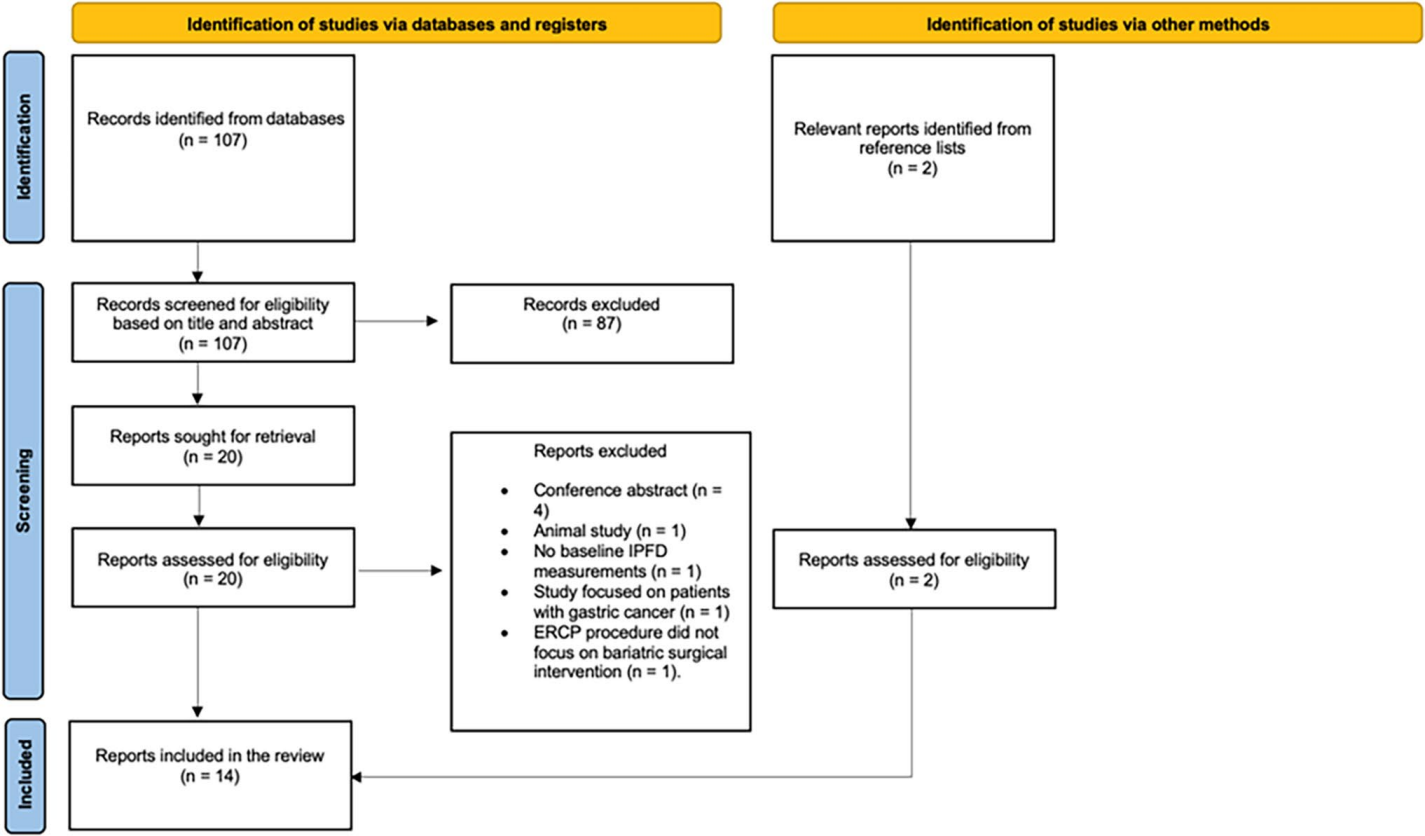
Study selection flow chart

After screening of 107 publications from the literature search and obtaining two publications from reference lists (Fig. 1), 14 studies met all the eligibility criteria [12–14, 17–27]. These included eight prospective and six retrospective cohort studies (Table 1). Most studies had a greater proportion of patients who had undergone SG than RYGB, with 11 out of the 14 studies having SG patients either exclusively or predominantly (Table 1). No study of endoscopic bariatric therapy was eligible. The quality assessment of the included studies in this systematic review generally showed robust methodology, with a mean score of 8.2 (and a standard deviation of 0.7) out of 9. Details are presented in Table S1. Of the included studies, MRI was the most frequently employed modality for the quantification of IPFD. Ten out of the 14 studies used chemical shift-encoded MRI, three studies—CT, and one study—magnetic resonance spectroscopy. Of the studies that used MRI, all but one study employed a 3 T scanner to generate proton density fat fraction maps (Table S2). Of the studies that used CT, all but one study determined IPFD by measuring the pancreas-to-spleen attenuation ratio (Table S3).

**Table 1:** Characteristics of the included studies

Study ID	Country	Study design	Method of IPFD measurement	Surgery (RGYB:SG)	Sex (M:W)	Mean age (years)	DM before surgery (*n*)	Mean weight before surgery (kg)
Gaborit et al. 2015 [12]	France	Prospective	MRS	7:13	6:14	43.3	8	119.9
Honka et al. 2015 [20]	Finland	Prospective	CT	8:15	0:23	45.0^a^ 41.0^b^	6	116^a^ 107^b^
Steven et al. 2016 (1) [25]	UK	Prospective	Chemical shift-encoded MRI	25:2	9:18	49.1^a^ 46.3^b^	18	121.1^a^ 114.5^b^
Steven et al., 2016 (2) [25]	UK	Prospective	Chemical shift-encoded MRI	8:1*	NR	NR	9	121.4
Umemura et al. 2017 [14]	Japan	Retrospective	CT	0:27	14:13	43.6	14	125.1
Lautenbach et al. 2018 [13]	Germany	Retrospective	Chemical shift-encoded MRI	11:0	2:9	43.5	1	136.5
Covarrubias et al. 2019 [24]	USA	Prospective	Chemical shift-encoded MRI	0:9	2:7	49.6	3	119
Hui et al. 2019 [22]	China	Prospective	Chemical shift-encoded MRI	2:8**	4:8	45.4	7	96.1
Kulali et al. 2019 [27]	Turkey	Retrospective	Chemical shift-encoded MRI	0:44	3:41	42	NR	NR
Salman et al. 2022 [21]	Egypt	Prospective	Chemical shift-encoded MRI	0:54	29:25	44.3	12	118.1
Bai et al. 2023 [17]	China	Retrospective	Chemical shift-encoded MRI	0:21	8:13	30	0	104.2
Cui et al. 2023 [18]	China	Prospective	Chemical shift-encoded MRI	0:49	18:31	31	NR	102.1
Yu et al. 2023 [23]	China	Retrospective	Chemical shift-encoded MRI	0:18	9:9	36.9	18	110.7
Hong et al. 2024 [19]	China	Retrospective	CT	0:32***	NR	NR	11***	NR

^a^Patients with diabetes mellitus before metabolic bariatric surgery; ^b^patients without diabetes mellitus before metabolic bariatric surgery

*One patient in this study did not get IPFD measured

**Two other patients underwent greater curvature plication

***Based on the subcohort with complete follow-up data

*CT* computed tomography, *DM* diabetes mellitus, *IPFD* intra-pancreatic fat deposition, *MRI* magnetic resonance imaging, *MRS* magnetic resonance spectroscopy, *NR* not reported, *RGYB* Roux-en-Y gastric bypass, *SG* sleeve gastrectomy

### Changes in IPFD and TPV After Metabolic Bariatric Surgery

**Fig. 2:**
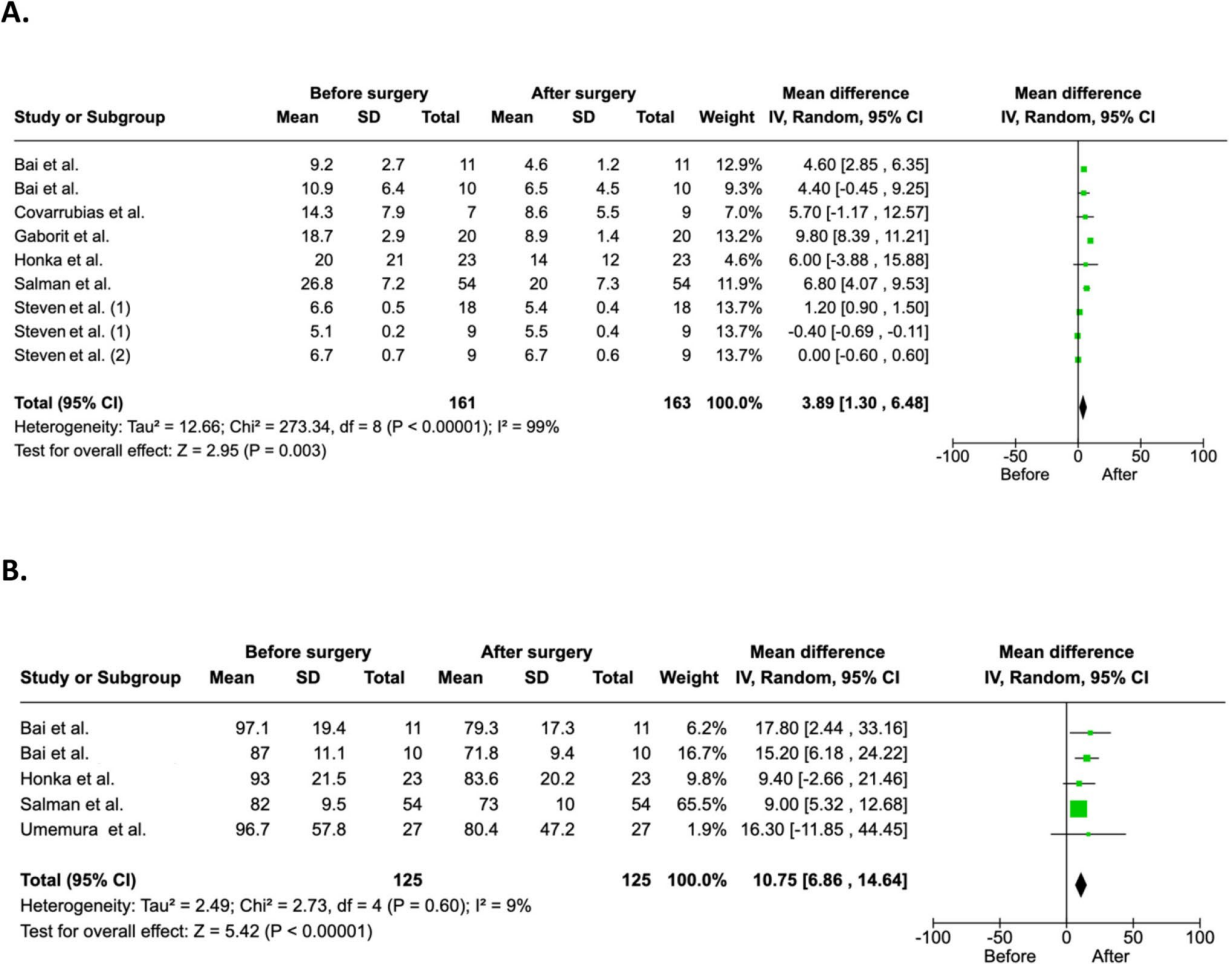
Changes in intra-pancreatic fat deposition (**A**) and total pancreas volume (**B**) from before to after metabolic bariatric surgery. Absolute changes are presented. The study by Bai et al. had two non-overlapping cohorts of patients: one cohort was investigated at baseline and three months after metabolic bariatric surgery, whereas the other cohort was investigated at baseline and 12 months or more after metabolic bariatric surgery [17]. The study by Stevens et al. presented data separately for patients with diabetes mellitus at baseline and those without it [25]. CI, confidence interval; IV, inverse variance; SD, standard deviation

Metabolic bariatric surgery led to a significant absolute reduction in IPFD of 3.89% (95% CI 1.30 to 6.48) (Fig. 2A). The mean relative reduction in IPFD by the end of the maximum follow-up period across the studies was 35.9%, with a standard deviation of 16.0%. Metabolic bariatric surgery led to a significant absolute reduction in TPV of 10.75 mL (95% CI 6.86 to 14.64) (Fig. 2B). Statistical heterogeneity for change in IPFD was high at *I*^2^ = 99%, whereas heterogeneity for change in TPV was low at *I*^2^ = 9%. Three studies also compared IPFD of patients after metabolic bariatric surgery versus healthy controls [12, 20, 23]. Two of them showed that IPFD had significantly reduced to the level of lean individuals [12, 23], whereas one study did not reach the predetermined level of statistical significance [20].

### Factors Affecting Change in IPFD After Metabolic Bariatric Surgery

**Fig. 3:**
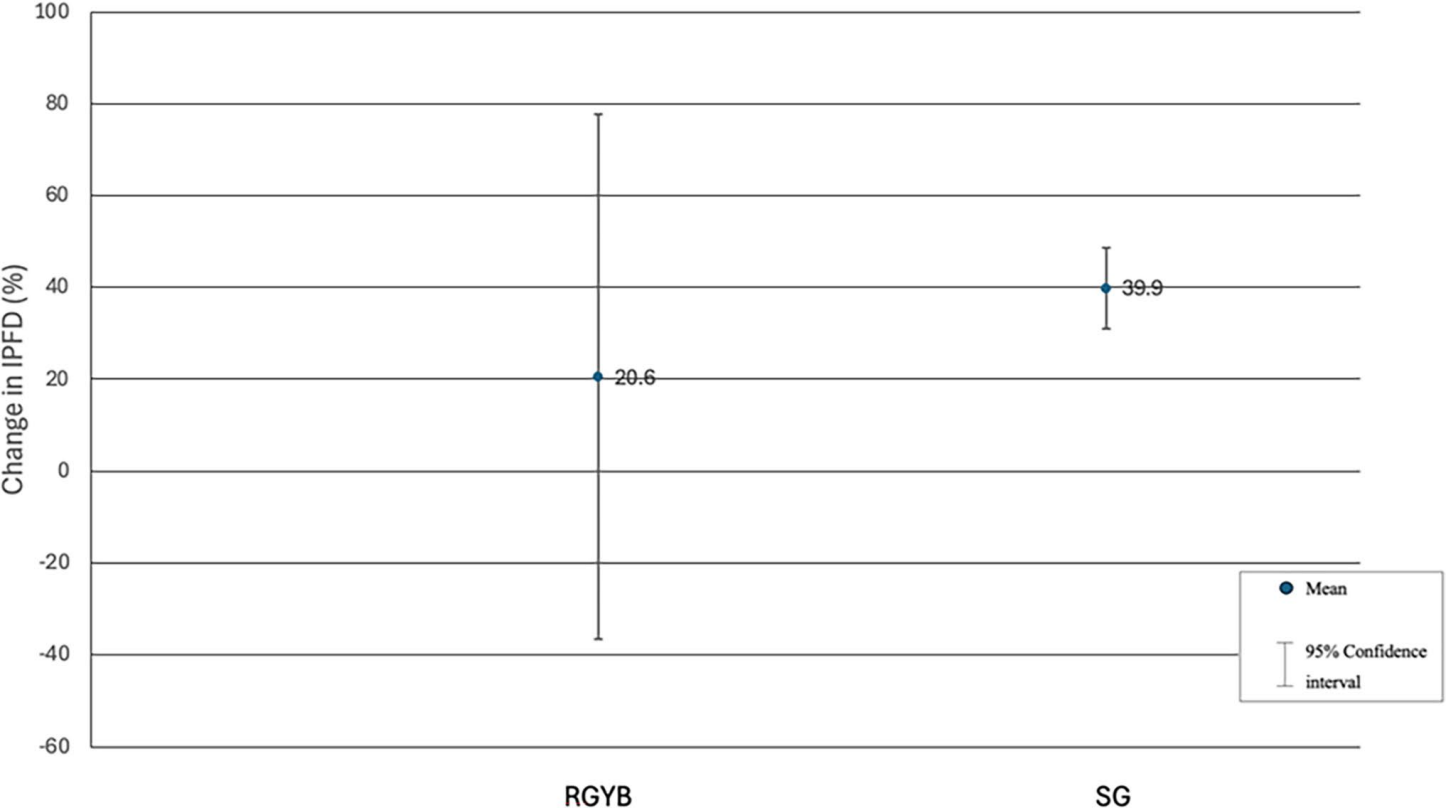
Changes in intra-pancreatic fat deposition according to the type of metabolic bariatric surgery used. Relative changes are presented. Studies were stratified according to the predominant surgery employed in each individual study. IPFD, intra-pancreatic fat deposition; RGYB, Roux-en-Y gastric bypass; SG, sleeve gastrectomy

**Fig. 4:**
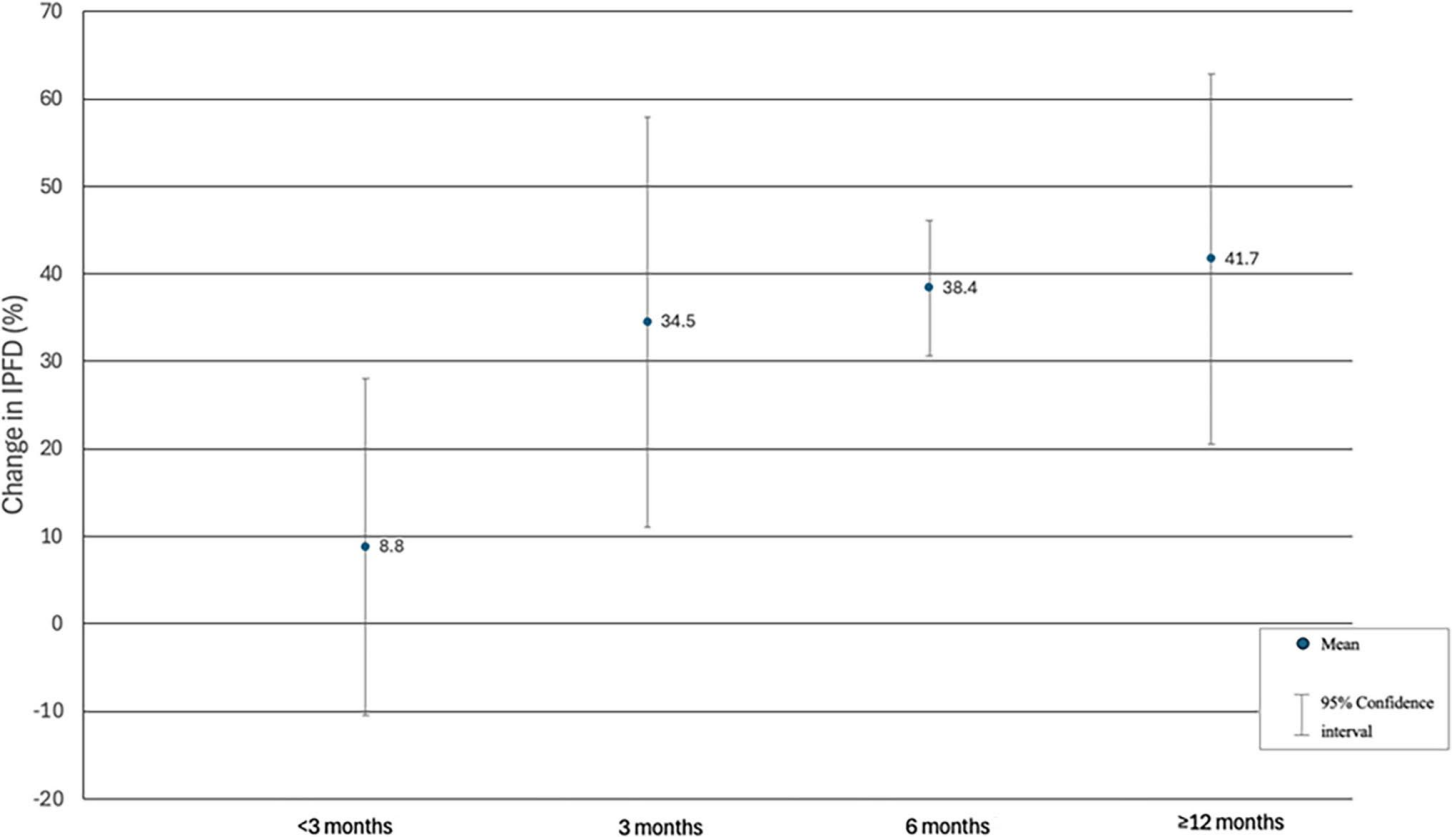
Changes in intra-pancreatic fat deposition according to the duration of follow-up. Relative changes are presented. Based on mean relative change in IPFD in each individual study. Data on all available follow-ups in each individual study are presented. IPFD, intra-pancreatic fat deposition

In the analysis comparing different types of surgery used, the mean relative change in IPFD showed no statistically significant difference between SG and RYGB (*p* > 0.05). The mean relative change for SG was 39.9% (95% CI 31.7 to 48.1), whereas the mean relative change for RYGB was 20.6% (95% CI − 36.5 to 77.7) (Fig. 3). In the analysis comparing different durations of follow-up, the mean relative change in IPFD was significantly lower (*p* < 0.05) at follow-ups of less than 3 months as compared with 6-month follow-up (Fig. 4). In the analysis comparing different imaging modalities used, the mean relative change in IPFD showed no statistically significant difference between CT and MRI (*p* > 0.05). The mean relative change for CT was 37.3% (95% CI 31.3 to 43.2), whereas the mean relative change for MRI was 34.3% (95% CI 19.8 to 48.9) (Fig. S1).

### Correlates of Change in IPFD After Metabolic Bariatric Surgery

**Fig. 5:**
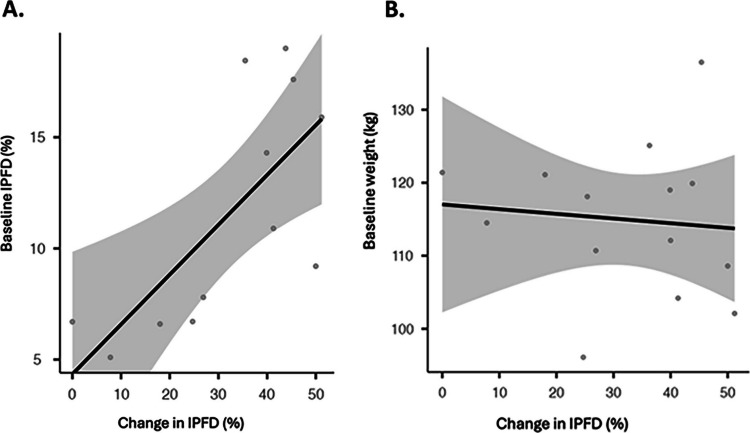
Correlations of changes in intra-pancreatic fat deposition with baseline IPFD (**A**) and baseline weight (**B**) in the included studies. Relative changes (more specifically, reductions) are presented. The solid line denotes the linear regression, whereas the shaded area denotes the 95% confidence interval. The 6-month follow-up data from the study by Hui et al. was used to provide a conservative estimate of change in intra-pancreatic fat deposition [22]. IPFD, intra-pancreatic fat deposition

**Fig. 6:**
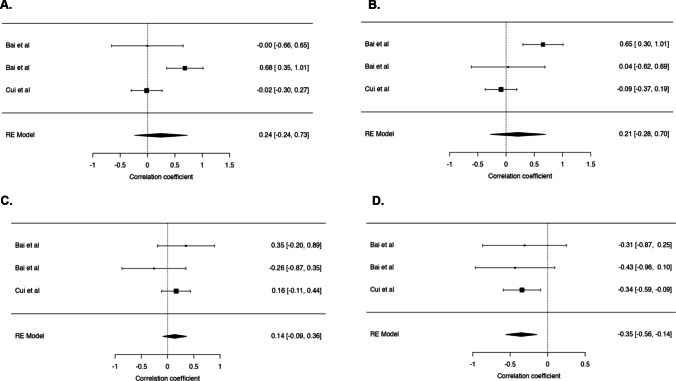
Correlations of changes in intra-pancreatic fat deposition with changes in homeostatic model assessment for insulin resistance (**A**), fasting insulin (**B**), fasting plasma glucose (**C**), and high-density lipoprotein cholesterol (**D**) in the included studies. Absolute changes are presented. The study by Bai et al. had two non-overlapping cohorts of patients: one cohort was investigated at baseline and 3 months after metabolic bariatric surgery, whereas the other cohort was investigated at baseline and 12 months or more after metabolic bariatric surgery [17]. RE, random effects

**Fig. 7:**
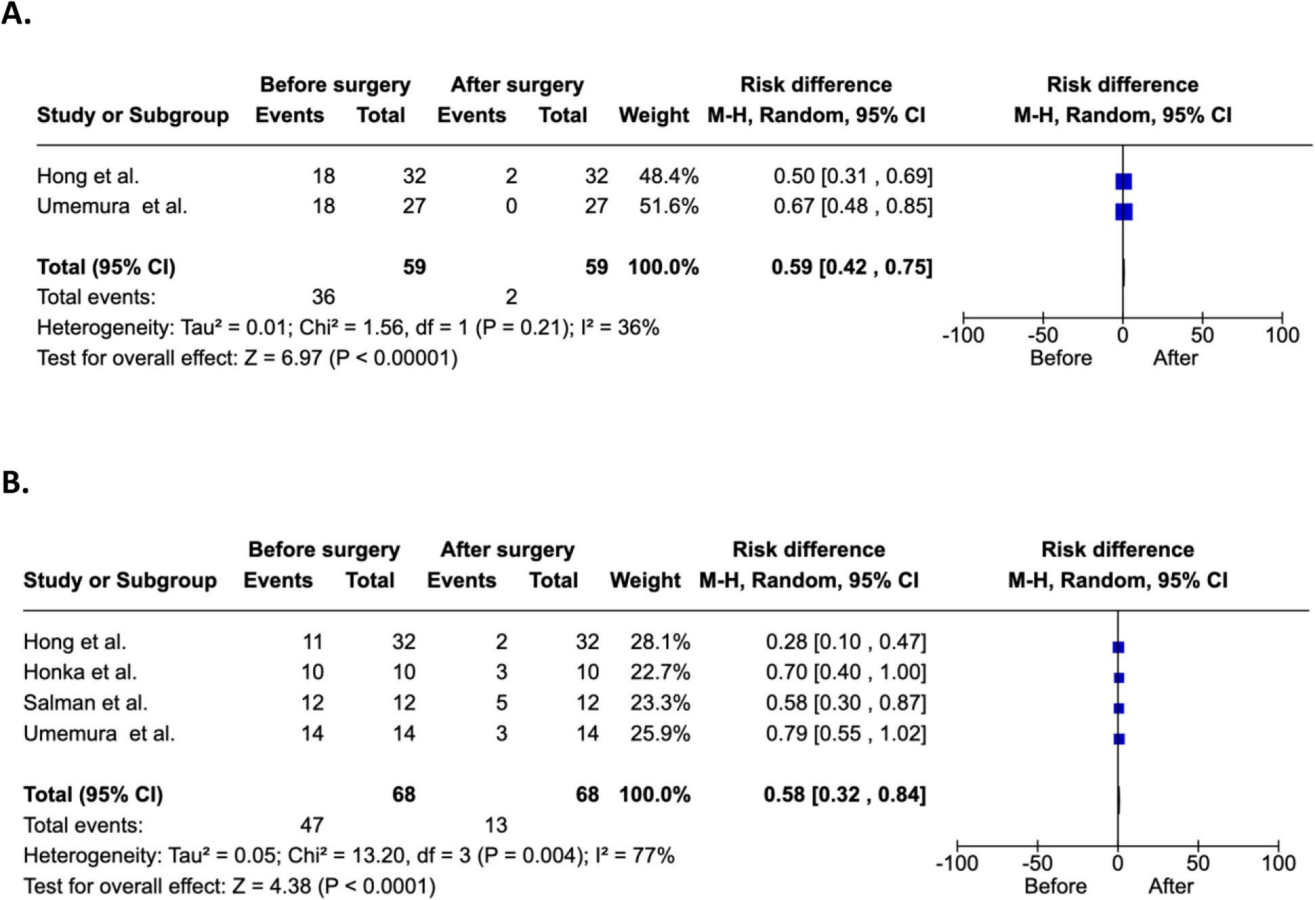
Changes in dyslipidaemia (**A**) and diabetes mellitus (**B**) status from before to after metabolic bariatric surgery. CI, confidence interval; M-H, Mantel–Haenszel

Change in IPFD after metabolic bariatric surgery was significantly positively correlated with baseline IPFD (*r* = 0.72; 95% CI 0.24 to 0.91; *p* = 0.009) (Fig. 5A). By contrast, change in IPFD was not significantly correlated with baseline weight (*r* =  − 0.10; 95% CI − 0.60 to 0.46; *p* = 0.742) (Fig. 5B). Change in IPFD after metabolic bariatric surgery was significantly negatively correlated with change in high-density lipoprotein cholesterol (*r* =  − 0.35; 95% CI − 0.56 to − 0.14; *p* < 0.001) (Fig. 6D). By contrast, change in IPFD was not significantly correlated with changes in fasting plasma insulin, fasting plasma glucose, and homeostatic model assessment for insulin resistance (Fig. 6A, B, C). The above correlations were accompanied by significantly reduced frequency of dyslipidaemia (*p* < 0.001) and diabetes mellitus (*p* < 0.001) following metabolic bariatric surgery in the included studies (Fig. 7A, B).

## Discussion

Metabolic bariatric surgery is well-known for its profound effects on weight loss and metabolic health. Its influence on morphology of the pancreas—particularly in relation to IPFD and, by extension, a broader biomarker TPV—has recently started to garner increasing interest due to potential implications for preventing pancreatic diseases. Several observational studies have demonstrated the protective effects of metabolic bariatric surgery on the pancreas. A 2016 cohort study by Krishna et al. reported that patients with acute pancreatitis who had undergone metabolic bariatric surgery had a 59% reduction in in-hospital mortality and shorter hospital stays (after accounting for associated medical problems and other covariates) [28]. A 2020 cohort study by Kröner et al. further supported these findings, showing a 53% reduction in in-hospital mortality and lower resource utilisation (after propensity matching) among patients with acute pancreatitis who had a prior history of metabolic bariatric surgery [29]. Further, a 2024 meta-analysis by Angelidi et al., including more than 3.7 million adults, found that metabolic bariatric surgery reduced the risk of developing pancreatic cancer by 54% [30]. A subgroup analysis revealed a remarkable 79% reduction in pancreatic cancer risk among individuals with type 2 diabetes [30]. Reductions in IPFD and TPV may not only serve as additional indicators for the success of bariatric procedures but also provide insights into their potential to reduce the risk of diseases of the pancreas [6]. The PANDORA (PANcreatic Disease Originating from intRa-pancreatic fAt) hypothesis advanced the field by postulating that IPFD plays a causative role in the development of diseases of the exocrine pancreas and endocrine pancreas alike [31]. This hypothesis has been bolstered by evidence from 2024 Mendelian randomisation studies demonstrating that higher IPFD was causally linked to increased risks of both pancreatic cancer and acute pancreatitis and chronic pancreatitis [32, 33]. A 2024 large prospective cohort study also found a significant association between IPFD at baseline and the future risk of type 2 diabetes [11]. By investigating the morphological changes within the pancreas following metabolic bariatric surgery, we therefore bridged the gap between clinical observations (such as reduced frequency of pancreatic diseases after metabolic bariatric surgery) and the PANDORA hypothesis.

For the first time, the present meta-analysis provided data on pooled absolute (3.9%) and relative (35.9%) IPFD reduction from before to after metabolic bariatric surgery. A 2017 meta-analysis by Singh et al. proposed a threshold of 6.2% for defining high IPFD [15], which suggests that an absolute reduction of 3.9% in the present study is a large magnitude of change that could be achieved by metabolic bariatric surgery. It is also worth noting that Bai et al. reported on the resolution of high IPFD in 75% of patients who had it before metabolic bariatric surgery [17]. Studies examining the effects of metabolic bariatric surgery in animal models have provided additional supporting evidence regarding its effect on IPFD. For instance, Rebours et al. demonstrated that IPFD and inflammation in Wistar rats with obesity decreased significantly and returned to near-normal levels in 3 months after SG or RYGB [34]. This finding suggests that metabolic bariatric surgery can reverse low-grade inflammation in the pancreas (a characteristic of high IPFD) and supports the notion that the reduction in reduced IPFD is a direct outcome of metabolic bariatric surgery [6, 31, 35]. Further, Otero et al. found that Wistar rats undergoing SG had significant reductions in IPFD and inflammatory cells in comparison with those subjected to merely caloric restriction [36]. The study also identified the downregulation of key lipogenic transcription factors (*Srebf1*, *Mogat2*, *Dgat1*) as contributors to reduced IPFD after SG. Moreover, the study found that guanylin peptides (that regulate fat metabolism and inflammation) were upregulated post-surgery [36]. Taken together, the above findings support metabolic bariatric surgery as an effective intervention for reducing IPFD and preventing the deleterious effects of high IPFD on pancreatic health.

In our analysis comparing the two main types of surgery, RYGB and SG, we found no statistically significant difference in IPFD reduction. However, the mean relative change in IPFD was notably higher in SG at 39.9%—nearly double that of RYGB at 20.6%. It is worth noting though that this difference might have been attributed to the limited number of RYGB studies and the fact that two of the three studies had a follow-up at less than 3 months after surgery [25, 26]. Although which procedure leads to the greatest reduction in IPFD cannot be determined conclusively based on our findings and this aspect requires additional studies, a meta-analysis by Angelidi et al. showed a potential advantage of SG over RYGB in reducing the risk of pancreatic cancer [30]. The authors demonstrated a lower risk of pancreatic cancer in patients who underwent SG (risk ratio of 0.24) than those who underwent RYGB (risk ratio of 0.52). The two main types of metabolic bariatric surgery also affect remission of type 2 diabetes, with our meta-analysis demonstrating a 42% lower frequency of type 2 diabetes after surgery (Fig. 7B). Although we were unable to investigate whether this effect is differential, an updated 2024 meta-analysis showed a significantly higher remission of type 2 diabetes after SG than RYGB (risk ratio of 1.15) [37]. Given that IPFD is a known precursor to pancreatic cancer [8, 38–40] and type 2 diabetes [11, 31], the above findings allude to the possibility that SG may result in greater IPFD reduction. Future research should validate our results in large prospective cohorts. Additionally, experimental research should clarify the distinct mechanistic pathways through which SG and RYGB influence IPFD and, by extension, risks of pancreatic diseases [31].

In analysing IPFD changes across different follow-up periods in the included studies, it was noted that substantial reductions in IPFD occurred at 3 months post-surgery and beyond, with a statistically significant difference between the less than 3 months and 6 months of follow-up. The nearly four-fold difference in IPFD reduction between follow-ups at less than 3 months and 3 months or more highlights the phase of ongoing fat reduction in the pancreas before the 3-month mark and the plateau phase between 3 and 12 months after the intervention. This suggests that, while immediate surgical effects like reduced caloric intake may benefit weight loss, the most substantial decrease in IPFD relies on metabolic and hormonal changes that gradually develop over several months [41, 42]. Similar timeline is observed in regard to the effect of medications on IPFD reduction [43]. Based on the above arguments, future clinical and research follow-ups using quantitative imaging should ideally occur at or after the 3-month mark, as this is when the largest IPFD reduction typically occurs.

The associations between changes in IPFD and metabolic parameters post-surgery observed in the present study highlight the nuanced interrelationship of fat in the pancreas with overall metabolic health. A significant correlation was found between IPFD reduction and improved lipid profile, specifically through increase in the circulating levels of high-density lipoprotein cholesterol. The frequency of dyslipidaemia was significantly reduced, too. In contrast, IPFD reduction did not significantly correlate with markers of glucose metabolism in the present study. These findings are in line with the results of recent large clinical studies on the primary effect of IPFD on lipid metabolism [44–52]. Also, we investigated the relationship between body composition at baseline and the extent of IPFD reduction. The results showed a strong positive correlation between IPFD at baseline and IPFD reduction during follow-up, suggesting that individuals with higher initial IPFD tend to have more substantial reductions in IPFD after the intervention. Notably, no significant correlation was observed between body weight at baseline and IPFD reduction, indicating that IPFD is not necessarily a function of weight [6, 15, 31].

Several limitations of the present study are to be acknowledged. First, the systematic review included a relatively small number of studies. However, this was the first systematic review on the subject and it was important to benchmark the published studies. Second, most studies did not include a lean control group for comparison. However, two studies did compare postoperative individuals with healthy controls and showed that IPFD reductions reached the levels observed in healthy controls [12, 23]. One study that did not find a statistical significance needs to be interpreted with caution as, unlike the other studies, healthy controls were not matched on sex with individuals undergoing metabolic bariatric surgery [20]. Third, nearly half of the included studies (6/14) were retrospective. However, the methodological quality across all included studies was high, with an average score of 8.2 on the Newcastle–Ottawa scale. Fourth, the statistical heterogeneity was very high (99%) in the primary analysis of IPFD and some secondary analyses presented very wide 95% CIs. These are not uncommon in pooled analyses of metabolic bariatric surgery as RYGB and SG are fundamentally dissimilar types of surgery, each with distinct mechanism of actions. Other contributing factors might have been variations in the duration of follow-up after metabolic bariatric surgery, variations in the MRI protocols used to determine IPFD, and differences in demographics as well as associated medical problems between participants in the included studies. The presented findings should be viewed as hypothesis-generating, leading to further purposefully designed studies exploring why some of the results show such high variation. Last, the present study did not account for possible confounding variables such as sex, age, and others [6, 31]. Future studies should control for these variables to yield more nuanced estimates of the effects of metabolic bariatric surgery on pancreas morphology.

## Conclusion

The present systematic review comprehensively investigated the effects of bariatric procedures on morphological changes in the pancreas and showed that metabolic bariatric surgery led to significant reductions in both IPFD and TPV. While no statistically significant differences were found between surgical techniques, SG tended toward greater IPFD reduction than RYGB. Further, the temporal aspect of IPFD reduction following metabolic bariatric surgery suggests a gradual response, with substantial reductions occurring no earlier than at 3 months after the intervention. These findings are aligned well with the PANDORA hypothesis, postulating that lowering IPFD may help prevent and alleviate the burden of both diseases of the exocrine pancreas and diseases of the endocrine pancreas.

## Supplementary Information

Below is the link to the electronic supplementary material.

Supplementary file1 (DOCX 80 KB)

## Data Availability

No datasets were generated or analysed during the current study.
