# Supplementary material for: The Effects of Metabolic Bariatric Surgery on Intra-pancreatic Fat Deposition and Total Pancreas Volume: a Systematic Review and Meta-analysis
Source: Obes Surg. 2025 Mar 17;35(4):1513–24. doi: 10.1007/s11695-025-07778-9 (PMC11976765; doi:10.1007/s11695-025-07778-9)
Supplement: Supplementary file 1 — Supplementary file1 (DOCX 80 KB) [file 11695_2025_7778_MOESM1_ESM.docx]

**Supplementary Table 1.** Methodological quality of the included studies

| Study ID | Selection | Comparability | Outcome | Overall score |
| --- | --- | --- | --- | --- |
| Gaborit et al., 2015 | 4 | 2 | 3 | 9 |
| Honka et al., 2015 | 4 | 2 | 3 | 9 |
| Steven et al., 2016 (1) | 4 | 2 | 3 | 9 |
| Steven et al., 2016 (2) | 3 | 2 | 2 | 7 |
| Umemura et al., 2017 | 3 | 2 | 3 | 8 |
| Lautenbach et al., 2018 | 3 | 2 | 3 | 8 |
| Covarrubias et al., 2019 | 3 | 2 | 3 | 8 |
| Hui et al., 2019 | 3 | 2 | 3 | 8 |
| Kulali et al., 2019 | 3 | 2 | 2 | 8 |
| Salman et al., 2022 | 3 | 2 | 3 | 8 |
| Bai et al., 2023 | 3 | 2 | 3 | 8 |
| Cui et al., 2023 | 3 | 2 | 2 | 7 |
| Yu et al., 2023 | 4 | 2 | 3 | 9 |
| Hong et al., 2024 | 4 | 2 | 3 | 9 |

**Supplementary Table 2.** Magnetic resonance imaging protocols used in the included studies

| Study ID | Radiofrequency coils used | Magnetic field strength | Type of scanner | Sequence used | Number of readers (experience) |
| --- | --- | --- | --- | --- | --- |
| Steven et al., 2016 (1) | 6-channel cardiac array or four large and medium flex surface coils | 3.0 T | Phillips Achieva | mDIXON | 1 |
| Steven et al., 2016 (2) | 6-channel cardiac array or four large and medium flex surface coils | 3.0 T | Phillips Achieva | mDIXON | Not reported |
| Lautenbach et al., 2018 | Not reported | 3.0 T | Phillips Ingenia | SS-FSE, respiratory-triggered T2WI, TSE, mDIXON | Not reported |
| Covarrubias et al., 2019 | 8-channel body array | 3.0 T | GE Signa EXCITE HDxt | SGRE, IDEAL-IQ | 2 (3 months; 1 year) |
| Hui et al., 2019 | 16-channel body array | 3.0 T | Philips Achieva X | mDIXON, STEAM | 2 |
| Kulali et al., 2019 | Not reported | 1.5 T | Siemens Magnetom Avanto | T2WI HASTE, T1WI | 2 |
| Salman et al., 2022 | Not reported | 3.0 T | Phillips Ingenia | SS-FSE, respiratory-triggered T2WI, mDIXON | 1 |
| Bai et al., 2023 | 8-channel body array | 3.0 T | GE Discovery MR 750 | T2WI, LAVA, IDEAL-IQ | 1 (6 years) |
| Cui et al., 2023 | Not reported | 3.0 T | Phillips INGENIA ELITION X | mDIXON | 1 (8 years) |
| Yu et al., 2023 | Not reported | 3.0 T | GE Discovery MR 750 | SS-FSE T2WI, GRE T1WI, IDEAL-IQ | 1 (5 years) |

*Abbreviations:* GRE= gradient echo; HASTE = half-Fourier acquisition single-shot turbo spin echo; IDEAL-IQ = iterative decomposition of water and fat with echo asymmetry and least squares estimation; LAVA = liver acceleration volume acquisition; SGRE = spoiled gradient echo; SS-FSE = single shot fast spin echo; STEAM = stimulated echo acquisition mode; T1WI = T1 weighted imaging; T2WI = T2 weighted imaging; TSE = transverse fast spin echo

**Supplementary Table 3.** Computed tomography protocols used in the included studies

| Study ID | Vendor | Type of scanner | Scanned region | Image post-processing |
| --- | --- | --- | --- | --- |
| Honka et al., 2015 | Phillips | Hybrid PET/ computed tomography (GE Discovery VCT) | Not reported | Carimas 2 software |
| Umemura et al., 2017 | Toshiba | 64-row computed tomography (Toshiba Aquilion) | Single slice at umbilicus | SYNAPSE VINCENT software |
| Hong et al., 2024 | Siemens | Dual source computed tomography (Siemens SOMATOM Definition Flash) | Apex of lungs to L3 vertebra | Mindways bone mineral density software |

*Abbreviation:* PET = positron emission tomography


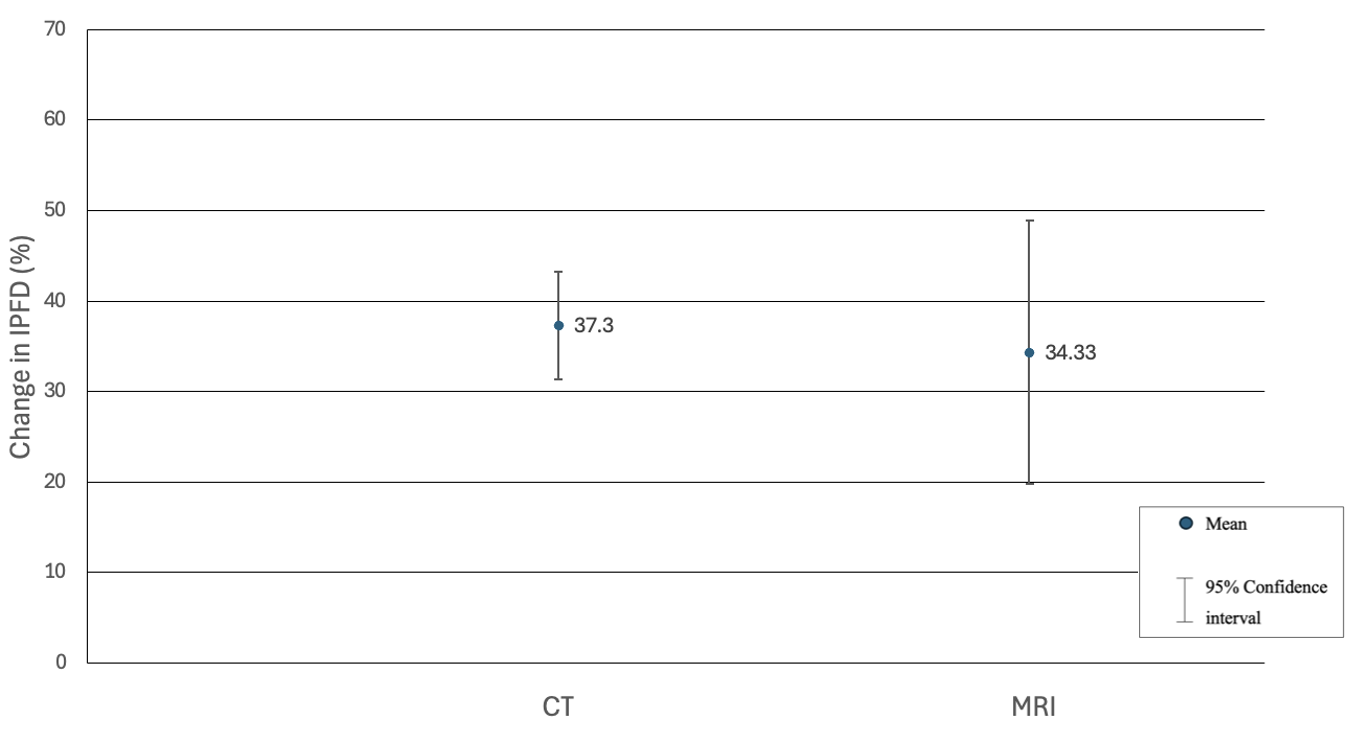


**Supplementary Figure 1.** Changes in intra-pancreatic fat deposition according to the imaging modality used.

*Footnote:* Relative changes are presented.

*Abbreviations:* CT= computed tomography; IPFD = intra-pancreatic fat deposition; MRI = magnetic resonance imaging
